# Editorial: Psychosocial drivers and outcomes of the cancer-related pain experience

**DOI:** 10.3389/fpsyg.2025.1680269

**Published:** 2025-09-09

**Authors:** Brennan P. Streck, Rachel D. Altshuler, Jeannine M. Brant, Cecile R. Buskbjerg, Kristine Kwekkeboom

**Affiliations:** ^1^Breast and Gynecological Cancer Research Group, Division of Cancer Prevention, National Cancer Institute, Rockville, MD, United States; ^2^City of Hope National Medical Center, Duarte, CA, United States; ^3^Department of Oncology, Aarhus University Hospital, Aarhus, Denmark; ^4^School of Nursing, University of Wisconsin-Madison, Madison, WI, United States

**Keywords:** cancer pain, psychosocial oncology, psycho-oncology, non-pharmacologic and pharmacologic management, pain interventions

## Introduction

Cancer-related pain, stemming from cancer and/or its treatments, is a widely reported symptom, with prevalence estimates ranging from 23 to 55% (Snijders et al., 2022). Severity and chronicity vary by cancer type, treatment, disease status, biological factors such as genetics, and psychosocial factors, and thus cancer pain represents a complex problem with multiple facets (Fillingim, 2017; Dalal and Bruera, 2012). To prevent and treat cancer-related pain, we must develop interventions that acknowledge and capitalize on the interplay between these components.

Interest in the relationship between psychosocial factors and cancer pain has persisted for decades because it provides an interventional target. By addressing psychosocial concerns, pain might be improved or prevented; likewise, effective pain management can improve psychosocial outcomes. Identifying, quantifying, and temporalizing relationships between psychosocial factors and cancer pain is a high priority for both the pain and psycho-oncology fields, as they are interrelated. This Research Topic includes four breaking research studies that identify psychosocial drivers and outcomes of the cancer-related pain experience, as well as a novel intervention that targets their interplay.

The first study examines how personality characteristics can affect the internalization and experience of cancer-related pain. Aho et al. describe how dimensions of personality interact with pain and psychological factors to affect quality of life.

As cancer survivors cope with chronic pain, their acceptance of or accommodation to pain as a new part of their lives can affect their daily functioning, health, and goals. However, many report being undertreated. A second study by Slaghmuylder et al. reports a qualitative analysis to understand survivors' accounts of their pain trajectories and the care they received.

In the third study, Klages et al. explore biopsychosocial risk factors associated with pain among recently diagnosed pediatric patients. Pediatric cancer pain is uniquely challenging to assess and treat due to developmental gaps and limited life experience of a young patient. Understanding associated psychosocial factors, such as the ones in this study, helps identify at-risk children and informs future trials.

Finally, LeBaron et al. address a clinical challenge in cancer pain: capturing a representative story of a person's pain experience between clinical visits. Because pain is highly dynamic, patients often struggle to summarize their pain experience over many days/weeks when asked for their pain level in clinic. LeBaron et al. discuss a novel remote health monitoring system designed to capture patients' and caregivers' pain experiences in real-time.

Collectively, these papers advance knowledge of psychosocial drivers and outcomes of cancer pain. We hope you enjoy this Research Topic.

## References

[B1] Dalal S. Bruera E. (2012). Assessing cancer pain. Curr. Pain Headache Rep. 16, 314–324. 10.1007/s11916-012-0274-y 22585314

[B2] Fillingim R. B. (2017). Individual differences in pain: understanding the mosaic that makes pain personal. Pain 158(Suppl. 1), S11–S18. 10.1097/j.pain.0000000000000775 27902569 PMC5350021

[B3] Snijders R. A. H. Brom L. Theunissen M. van den Beuken-van Everdingen M. H. J. (2022). Update on prevalence of pain in patients with cancer. Cancer 15:591. 10.3390/cancers15030591 36765547 PMC9913127

